# Prevalence of tick-borne pathogens in ixodid ticks collected from domestic animals in southeast Iran

**DOI:** 10.1016/j.nmni.2026.101769

**Published:** 2026-05-21

**Authors:** Shahin seidi, Mina Latifian, Safoura Moradkasani, Ali Ghorbani, Mina Arabian, Mohammad khalili, Saber Esmaeili

**Affiliations:** a Department of Epidemiology and Biostatics, Research Centre for Emerging and Reemerging Infectious Diseases, WHO Collaborating Center for Vector-Borne Diseases, Pasteur Institute of Iran, Tehran, Iran; b National Reference Laboratory for Plague, Tularemia and Q fever, Research Centre for Emerging and Reemerging Infectious Diseases, Pasteur Institute of Iran, Akanlu, Kabudar Ahang, Hamadan, Iran; c Department of Pathobiology and Clinical Sciences, School of Veterinary Medicine, Shahid Bahonar University of Kerman, Kerman, Iran

**Keywords:** *Rickettsia*, *Ehrlichia*, Ixodid ticks, Iran, *Coxiella burnetii*

## Abstract

**Background:**

Tick-borne pathogens (TBPs) pose a significant threat to livestock production in Kerman Province, Iran. Despite the high prevalence of TBPs, our understanding of specific pathogens circulating within local tick populations remains limited. Furthermore, the potential for co-infections with multiple microorganisms complicates the diagnosis and disease management. This study aimed to investigate the prevalence of TBPs in ticks collected from domestic animals in Kerman province using real-time quantitative PCR (qPCR).

**Methods:**

Tick collection was conducted between April and June 2022 from 199 domestic animals (63 cattle, 63 sheep, 63 goats, and 10 dogs) across 65 villages in three counties of Kerman Province, Iran. Collected ticks were subjected to qPCR assays targeting *Coxiella burnetii*, *Bartonella* spp., *Rickettsia* spp., *Francisella* spp., *Borrelia* spp., and *Ehrlichia* spp. Phylogenetic analysis of the amplified DNA sequences was performed to elucidate the genetic relationships among the detected pathogens.

**Results:**

In this study, 707 ixodid ticks was collected included *Hyalomma marginatum marginatum* (70.7%, 108 pools) and *Rhipicephalus linnaei* (29.2%, 27 pools). Based on molecular analysis, *Rickettsia* spp., *Ehrlichia* spp, *C. burnetii* and *Bartonella* spp. were detected in 55.5%, 9.6%, 8.1% and 0.7%, respectively. *Rickettsia aeschlimannii*, *R. sibirica*, *R. conorii* subsp. *israelensis* and *R. africae* were identified based on sequencing and phylogenetical analysis.

Co-infections were also observed: 5.9% of the pools (8 pools) were co-infected with *Ehrlichia* spp. and *Rickettsia* spp., 3.7% (5 pools) with *Rickettsia* spp. and *C. burnetii*, and 0.7% (1 pool) with *Rickettsia* spp. and *Bartonella* spp. No infections with *Francisella* spp. or *Borrelia* spp. were detected.

**Conclusions:**

This study demonstrates the presence of multiple tick-borne pathogens of veterinary and public health significance in ruminants from Kerman Province, highlighting the need for further research on tick-borne diseases affecting both animal and human populations in this and surrounding regions.

## 1 Introduction

Ticks are obligate, blood-feeding ectoparasites that infest a wide range of vertebrate hosts, including humans [1]. These tiny arachnids are found in diverse habitats worldwide and pose a significant health and economic threat to the global animal industries [[2], [3], [4]]. Ticks can directly harm hosts by, causing stress, irritation, allergy, anemia, weight loss, and paralysis. Indirectly, they transmit various pathogens, including bacteria, fungi, protozoa, rickettsia, spirochetes, and viruses [[1], [2], [3]]. Among these pathogens, *Bartonella* spp., *Coxiella burnetii*, *Ehrlichia canis*, *Rickettsia* spp., and *Francisella tularensis* are of particular concern in human and veterinary medicine [5,6]. These agents cause substantial health problems and economic losses, particularly in livestock, and in subtropical and tropical regions [1]. Furthermore, ongoing climate and seasonal changes are believed to contribute to the emergence and re-emergence of ticks and tick-borne diseases in both animals and humans [7].

Iran, with its diverse climate and large geographical area, is susceptible to a variety of tick-borne diseases [[8], [9], [10]]. The country's heavy reliance on livestock, particularly small ruminants, for rural sustenance exacerbates the problem [11]. Kerman Province, the largest province in Iran, faces significant challenges due to poor infrastructure, limited resources, and inadequate veterinary services. Therefore, TBDs pose a major health threat to humans and animals in this region [9,12]. TBDs significantly hinder livestock health and productivity in Iran, which is a primary source of income for Iranian farmers. *Hyalomma* spp., and *Rhipicephalus* spp. are the primary vectors of these diseases in the country [13].

The genus *Rickettsia* consists of obligate intracellular, Gram-negative, arthropod-borne bacteria responsible for a variety of diseases in humans and livestock [14]. With over 35 species, *Rickettsia* causes emerging and re-emerging infectious diseases worldwide [15]. These pathogens are categorized into four main groups based on serological and genomic characteristics [16]. More than 15 *Rickettsia* spp. are human pathogens, causing diseases that range from mild to severe [17]. The geographic expansion of tick populations, driven by climate change, has increased the incidence of rickettsial infections [9], particularly in regions like the Mediterranean, including Iran [13]. Recently, cases of Mediterranean spotted fever (*R. conorii*) have been misdiagnosed as Crimean-Congo hemorrhagic fever in Iran, indicating a growing public health concern [18]. Additionally, recent studies have identified *Rickettsia* species in ticks, further emphasizing the need for better surveillance and control measures [9].

Another important tick-borne disease is ehrlichiosis, caused by *Ehrlichia* spp. *E. chaffeensis* and *E. ewingii* cause human monocytic and granulocytic ehrlichiosis [19,20], respectively, leading to symptoms such as fever, myalgia, and severe complications in immunocompromised individuals [[21], [22], [23]]. While *Ehrlichia* spp. have been detected in Iranian livestock, their presence in tick vectors remains underexplored. Additionally, *Q fever*, caused by the rickettsia-like bacterium *C. burnetii*, is a zoonotic disease affecting both humans and animals, particularly livestock workers [24]. The disease is primarily transmitted through inhalation of infectious agents, consumption of unpasteurized dairy, and contact with infected tissues [25], Although tick bites are considered a secondary route of transmission, they may play a role in maintaining the pathogen in nature, necessitating further investigation [24,26].

Tick-borne pathogens (TBPs) in domestic animals have been extensively studied in various parts of Iran [[8], [9], [10], [11]]. Recent advances in molecular techniques have revealed a diverse array of TBPs infecting livestock in the country. However, research on TBPs in the Kerman Province remains limited, with a primary focus on *Rickettsia* spp. [9,27]. This study aimed to address this knowledge gap by employing qPCR to investigate the molecular epidemiology and prevalence of various microorganisms, including co-infections, in hard ticks parasitizing domestic animals across three cities in the Kerman Province, Iran.

## 2 Materials and methods

### 2.1 Ethics Statement

This study was conducted in accordance with the ethical standards of Kerman University of Medical Sciences (Ethics Approval Code: IR.KMU.AEC.1404).

### 2.2 Study area and ticks sampling

**Fig. 1:**
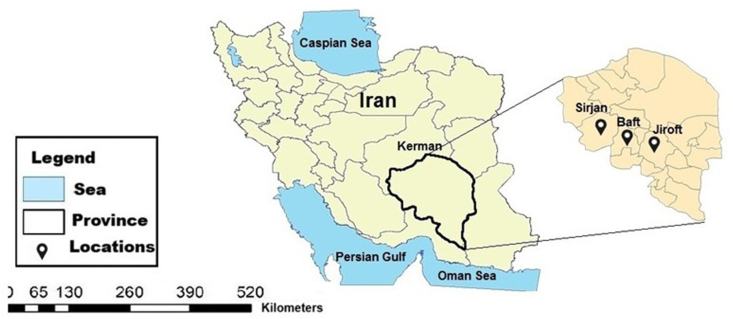
Map of Iran showing the districts included in this study. The names of the districts include Sirjan, Baft, and Jiroft, from Kerman Province.

This study was conducted in three cities within Kerman Province: Baft, Jiroft, and Sirjan. Situated in southeastern Iran on the central plateau, Kerman Province spans a vast area of over 183,285 square kilometers, constituting approximately 11% of the country's total land mass. With a rich history dating back to 4,000 BC, Kerman is Iran's largest province, geographically positioned between 53°26′ and 59°29′ east longitude and 25°55′ and 32° north latitude. Baft, a mountainous city characterized by a cold climate, is located in southwestern Kerman at an altitude of nearly 2,300 m above sea level. According to the 2016 Iranian census, the population was 34,517. Sirjan County, another southwestern region of Kerman, is home to 245,203 people. Finally, Jiroft County, situated in the southern part of the province, boasts a population of 191,460 and is recognized as a significant urban center in Kerman (Fig. 1).

A total of 707 (135 pools) Ixodid ticks were collected from 199 animals (sheep, goats, cattle, and dogs) across 24 villages in Baft County, 23 villages in Sirjan County, and 23 villages in Jiroft County, Kerman Province, Iran, between April and June 2022 (Fig. 1; Table 3). Ticks were manually removed from various body parts, including the ears, neck, and perineal region, and preserved in 70% ethanol in individually labeled Eppendorf tubes.

### 2.3 Morphological identification of ticks and DNA extraction

Ticks were morphologically identified using a dissecting microscope (Olympus SZ40, Olympus Corporation, Tokyo, Japan) and a taxonomic key specific to Iranian ticks [28]. Subsequently, DNA was extracted from 108 pools *H. marginatum marg*inatum and 27 pools of *Rh. linnaei* collected from three city districts in Kerman Province. Total genomic DNA was extracted from tick samples using a commercial DNA extraction kit (Zybio Nucleic Acid Extraction Kit; Zybio Inc., China). Briefly, tick samples were homogenized in Tissue lyzer II (Qiagen, Hilden, Germany) using a lysis buffer. The homogenate was then subjected to DNA extraction, according to the manufacturer's protocol. The extracted DNA was stored at −20 °C until further analysis.

### 2.4 Real-time quantitative PCR (qPCR)-Based detection of tick-borne microorganisms

Specific primers (Table 1) were designed to detect *C*. *burnetii*, *Bartonella* spp., *Rickettsia* spp., *Francisella* spp., and *Borrelia* spp. qPCR was performed using a Corbett Rotor-Gene 6000 thermocycler (Corbett Life Science, Germany) with the following reaction conditions: 4 μL DNA template, 10 μL 2X Maxima Probe qPCR Master Mix (Amplicon, Denmark), 1 μL of each primer (10 μM), 0.2 μL probe (10 μM), and 4.5 μL nuclease-free water for a final volume of 20 μL. The thermal cycling profile consisted of an initial denaturation step at 95 °C for 10 min, followed by 45 cycles at 95 °C for 15 s and 60 °C for 60 s. *R. conorii* genomic DNA (Vircell, Germany) served as a positive control for the *Rickettsia* spp. *F. tularensis* subsp*. holarctica* NCTC 10857 was used as a positive control for *Francisella* spp.

**Table 1:** Oligonucleotide sequences of primers and probes used in this study.

Organism	Gene target	Primer	Sequence (5′- 3′)	Reference
*Coxiella burnetii*	*IS1111*	Forward	5′-AAAACGGATAAAAAGAGTCTGTGGTT-3′	[29]
		Reverse	5′-CCACACAAGCGCGATTCAT-3′	
		Probe	5′-6-FAM-AAAGCACTCATTGAGCGCCGCG-TAMRA-3′	
*Rickettsia* spp.	*16S rRNA*	Forward	5′-CGCAACCCTYATTCTTATTTGC-3′	[30]
		Reverse	5′-CCTCTGTAAACACCATTGTAGCA-3′	
		Probe	5′-6- FAM-TAAGAAAACTGCCGGTGATAAGCCGGAG–TAMRA-3′	
*Bartonella* spp.	*16S rRNA*	Forward	5′-TTAGAGTGAGCGGCAAAC-3′	[31]
		Reverse	5′-TACCGTCATTATCTTCACCG-3′	
		Probe	5′-6FAM'-GGAGGGCTTGTAGCTCAGYTGGTTAGAGCG_TAMRA-3′	
*Ehrlichia* spp.	*16S rRNA*	Forward	5′-TCGCTATTAGATGAGCCTACGT-3′	[32]
		Reverse	5′-GAGTCTGGACCGTATCTCAGT-3′	
*Borrelia* spp.	*16S rRNA*	Forward	5′-GGTCAAGACTGACGCTGAGTCA-3′	[33]
		Reverse	5′-GGCGGCCACTTAACACGTTAG-3′	
		Probe	5′-Fam-TCTACGCTGTAAACGATGCACACTTGGTG-BHQ-1-3′	
*Francisella tularensis*	*ISftu2*	Forward	5′- TTGGTAGATCAGTTGGTAGGATAACC-3′ TTGGTAGATCAGTTGGTAGGATAACC-3 TTGGTAGATCAGTTGGTAGGATAACC-3	[34]
		Reverse	5′-TGAGTTTTATCCTCTGACAACAATATTTC-3′	
		Probe	5′6-Fam-AAAATCCATGCTATGACTGATGCTTTAGGTAATCCA-TAMRA-3′	

A SYBR Green qPCR assay targeting the *16S rRNA* gene was used to detect *Ehrlichia* spp. (Table 1). The 20 μL reaction mixture consisted of 10 μL of Cyber Green Plus 2x Master Mix Green Low ROX™ (AmpliQon, Denmark), 700 nM of each primer, 4 μL of template DNA, and nuclease-free water. Amplification was performed on a Corbett Rotor-Gene 6000 system (Corbett Life Science, Australia) under the following thermal cycling conditions: an initial denaturation step at 95 °C for 10 min, followed by 40 cycles of denaturation at 95 °C for 15 s, annealing at 60 °C for 20 s, and extension at 72 °C for 20 s. Melting curve analysis was performed to confirm the specificity of the PCR products. Data analysis was performed using the Rotor-Gene Q Series Software.

### 2.5 Identification of *Rickettsia* and *Ehrlichia* spp

Samples with Ct values below 30 in the initial qPCR screening were selected for further species identification. To achieve this, PCR amplification was performed targeting the *gltA* gene (830 bp) for *Rickettsia* spp., the *ompA* gene (632 bp) for *R. africae*, and the *16S rRNA* gene (555 bp) for *Ehrlichia* spp. isolated from *H. marginatum marginatum* and *Rh. linnaei*. Each 20 μL reaction contained 10 μL of TEMPase Hot Start Master Mix Blue (Ampliqon, Denmark), 4 μL of template DNA, 5 μL of deionized water, and 0.5 μM of each specific primer. PCR amplification was performed using the primers and thermal cycling conditions outlined in Table 2. PCR products were then separated via 1% agarose gel electrophoresis. Amplicons of the expected band sizes were subsequently purified and sequenced by Genomin Co. (Tehran, Iran).

**Table 2:** Primer sequences used for species determination using PCR method in this study.

Organism	Gene target	Sequence (5′- 3′)	Amplicon size (bp)	Thermal conditions	Reference
*Rickettsia* spp.	*glt*A	Forward: 5′- GCTCTTCTCATCCTATGGCTATTAT -3′ Reverse: 5′- CAGGGTCTTCRTGCATTTCTT -3′	575	one cycle of 5 min at 95 °C, 40 cycles of 30 s at 94 °C, 30 s at 58 °C, and 1 min at 72 °C, with a final step of 7 min at 72 °C.	[35]
	*ompA*	Forward: 5′- ATGGCRAATATTTCTCCAAAA -3′ Reverse: 5′- GTTCCGTTAATGGCAGCATCT -3′	632	one cycle of 5 min at 95 °C, 40 cycles of 30 s at 95 °C, 30 s at 55 °C, and 1 min at 72 °C, with a final step of 10 min at 72 °C.	[36]
*Ehrlichia* spp.	*16S rRNA*	Forward: 5′ -CTAGAGGTCGAAAGAGGATAG-3′ Reverse: 5′-GTGCTGATTTGACATCATCC-3′	555	one cycle of 5 min at 95 °C, 40 cycles of 20 s at 95 °C, 40 s at 56 °C, and 45 s at 72 °C, with a final step of 5 min at 72 °C.	[37]

**Table 3:** Details of ticks of domestic animals from the Kerman of Iran used in this study.

	Host				Ticks: (Female/Male)		
Location	Sheep	Goat	Dog	Cattle	*Hyalomma marginatum marginatum.*	*Rhipicephalus linnaei*	Total Ticks(F/M)
Baft	20	20	5	20	49 pool (135/131)	-	49 pool (135/131)
Sirjan	23	23	1	23	45 pool (57/149)	2 pool (6/0)	47 pool (63/149)
Jiroft	20	20	4	20	14 pool (13/15)	25 pool (90/111)	39 pool (103/126)
Total	63	63	10	63	108 pool (205/295)	27 pool (96/111)	135 pool (301/406)

### 2.6 Phylogenetic analysis

Following amplification and gel electrophoresis, 19 amplicons with expected band sizes were purified and sequenced. Chromatograms were analyzed using Chromas Lite v2.01 software (Technelysium Pty Ltd, Australia) to ensure data quality. Raw sequences from both the forward and reverse strands were obtained to minimize errors. Complementary strands of each sequenced product were edited and assembled using MEGA X software (Molecular Evolutionary Genetics Analysis, Version X) [38]. The resulting 19 high-quality sequences were deposited in the National Center for Biotechnology Information (NCBI) GenBank database, and accession numbers were acquired. Sequence similarity searches were performed using the Basic Local Alignment Search Tool (BLAST) in NCBI to identify closely related sequences within the database. Additionally, a phylogenetic tree was constructed using the MEGA X software employing the maximum likelihood method and bootstrapping with 1000 replicates. This analysis aimed to elucidate the evolutionary relationships among the sequenced amplicons.

### 2.7 Statistical analysis

Data were analyzed using IBM SPSS Statistics software, version 26. Descriptive statistics were presented as frequencies and percentages. The association between infection status and categorical variables was evaluated using the Chi-square test or Fisher's exact test, as appropriate. Univariate logistic regression analysis was performed to estimate odds ratios (ORs) and corresponding 95% confidence intervals (CIs). A p-value of less than 0.05 was considered statistically significant …

## 3 Results

**Fig. 2:**
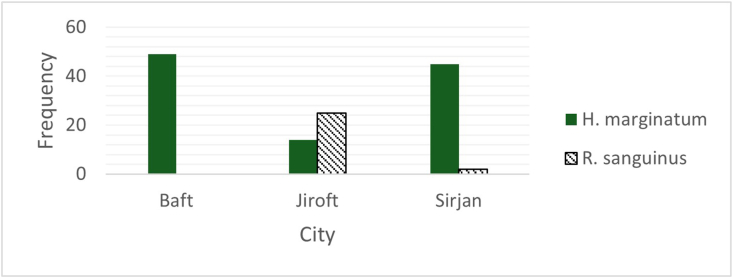
Geographic distribution of tick pool in various cities.

**Fig. 3:**
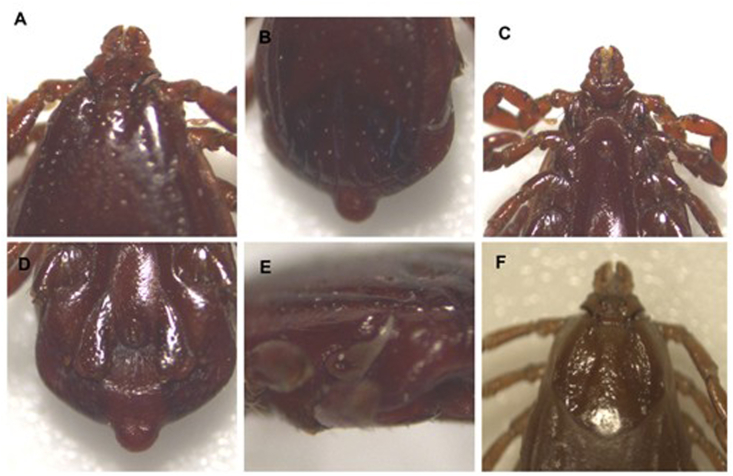
Morphological specific characteristics of *Rhipicephalus linnaei* (male/female). (A) Basis capituli with lateral angles blunt, interstitial punctation size minute to small, interstitial punctuation distribution dense, and setiferous punctations distinct. Cervical fields without depression apparent and texture wrinkles. (B) Conscutum color dark. Lateral grooves with a distinct groove and texture distinctly punctate. (C) Anterior spurs coxa I without visible dorsally. (D) Males with broad adanal plates and caudal appendages. (E) The spiracle plate narrow tail, half the width of adjacent festoon. (F) Female with lateral angles of basis capituli blunt. Cervical fields large and curved. Scutum posterior margin slightly sinuous.

**Fig. 4:**
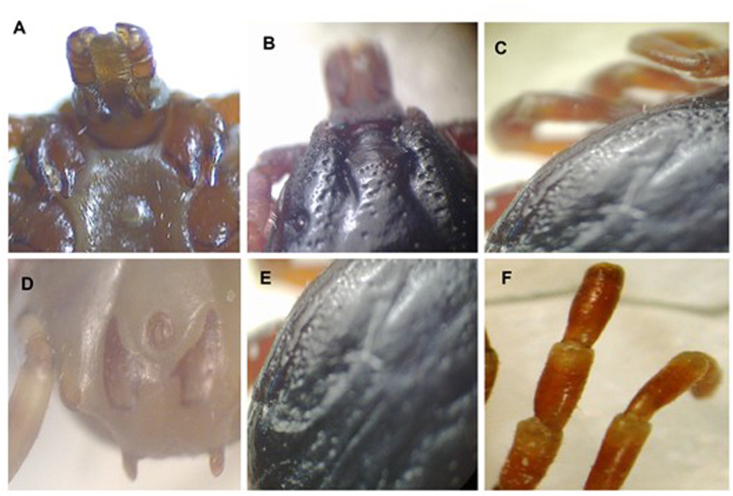
Morphological specific characteristics of *Hyalomma marginatum marginatum* (male). (A) Internal and external spurs as well as fissure of coxa I long. (B) Cervical field whith a depression apparent. The eyes convex. (C) Lateral grooves long (they continue toward eyes as lines of punctations). (D) Adanal plates whith square end. Subanal plate alignment with adanal plates. Subanal plates distinct (but small). (E) Consultum dark colored whith posteromedian groove. (F) Leg colouration with pale rings.

A total of 707 (135 pools) live ticks were collected from the body surfaces of various animals, including goats (n = 63), sheep (n = 63), cattle (n = 63), and dogs (n = 10) in Kerman Province, Iran. Samples were gathered from 21 villages in Baft County (49 pools), 24 villages in Sirjan County (47 pools), and 20 villages in Jiroft County (39 pools) (Fig. 2 & Table 3). Tick identification was conducted using a validated identification key for the Iranian ticks (Fig. 3, Fig. 4). *H. marginatum marginatum* was the most prevalent tick species, accounting for 500 samples (70.7%; 95% CI: 66.74%–74.70%) of the total. *Rh. linnaei* showed the lowest prevalence, with 207 samples (29.2%; 95% CI: 25.92%–32.62%). A significant difference in the infection rates of *H. marginatum marginatum* and *Rh. linnaei* was observed in Baft (P < 0.0084) and Sirjan (P < 0.0314) counties.

### 3.1 Morphologic results

All *Rh. linnaei* specimens from Kerman showed the specific morphological characteristics expected for this species (Fig. 3) [28].

All *H. marginatum marginatum* specimens from Kerman showed the specific morphological characteristics expected for this species (Fig. 4) [28].

### 3.2 Pathogens detection

A total of 135 tick pools, consisting of 108 *H. marginatum marginatum* and 27 Rh. *linnaei*, were collected from livestock in Kerman province and screened for a variety of tick-borne pathogens. While no infections with *F. tularensis* and *Borrelia* spp. were detected, a significant proportion of the ticks were found to be infected with at least one pathogen. A total of 86 out of 135 tested pools (63.7%) were infected with at least one pathogen. Specifically, 70 pools (64.8%; 95% CI: 55.8%–73.8%) of *H. marginatum marginatum* and 16 pools (59.2%; 95% CI: 40.7%–77.8%). of *Rh. linnaei* tested positive for pathogens.

Among the pools, 75 (55.5%) tested positive for *Rickettsia* species. Infection was detected in 65 pools of *H. marginatum* (60.2%) and 10 pools of *R.linnaei* (37.0%), with a statistically significant difference between tick species (OR = 2.93; 95% CI = 0.86–10.01; P = 0.03). Species-level identification was achieved for 18 positive pools. The dominant species was *R. aeschlimannii*, detected in 13 *H. marginatum* pools (72.2%), while none were found in *R. linnaei*. Most *R. aeschlimannii* infections were recorded in Sirjan (9 pools) and Baft (4 pools). Additionally, one *R. linnaei* pool from Jiroft was positive for *R. conorii* subsp. *israeliensis* (5.5%). Four *H. marginatum* pools from Sirjan were positive for other *Rickettsia* spp, including *R. sibirica* (3 pools, 16.6%) and *R. africae* (1 pool, 5.5%). The prevalence of *Rickettsia* infection did not differ significantly among cities (Baft: 34.7%; Jiroft: 24.0%; Sirjan: 41.3%; P = 0.373). Overall, *Rickettsia* spp. were widely distributed across tick species and regions, with the highest prevalence in *H. marginatum* and in Sirjan.

Thirteen pools (9.6%) were positive for *Ehrlichia* spp., including 12 *H. marginatum* pools (11.1%) and one *R. linnaei* pool (3.7%). Although the prevalence was higher in *H. marginatum*, the difference was not statistically significant (OR = 3.41; 95% CI = 0.42–27.45; P = 0.47). The highest infection rate was recorded in Baft (14.3%), followed by Jiroft (10.0%) and Sirjan (4.3%).

Eleven pools (8.1%) were positive for *C. burnetii*, including three *H. marginatum* pools (2.8%) and eight *R. linnaei* pools (29.6%). The probability of *C. burnetii* infection was significantly higher in *R. linnaei* than in *H. marginatum* (OR = 0.07; 95% CI = 0.02–0.30; P = 0.001). *C. burnetii* was detected exclusively in samples collected from Jiroft (27.5%), although no significant association was observed between tick species and collection site. In addition, one pool of *H. marginatum marginatum* (0.9%) tested positive for *Bartonella* spp.

**Fig. 5:**
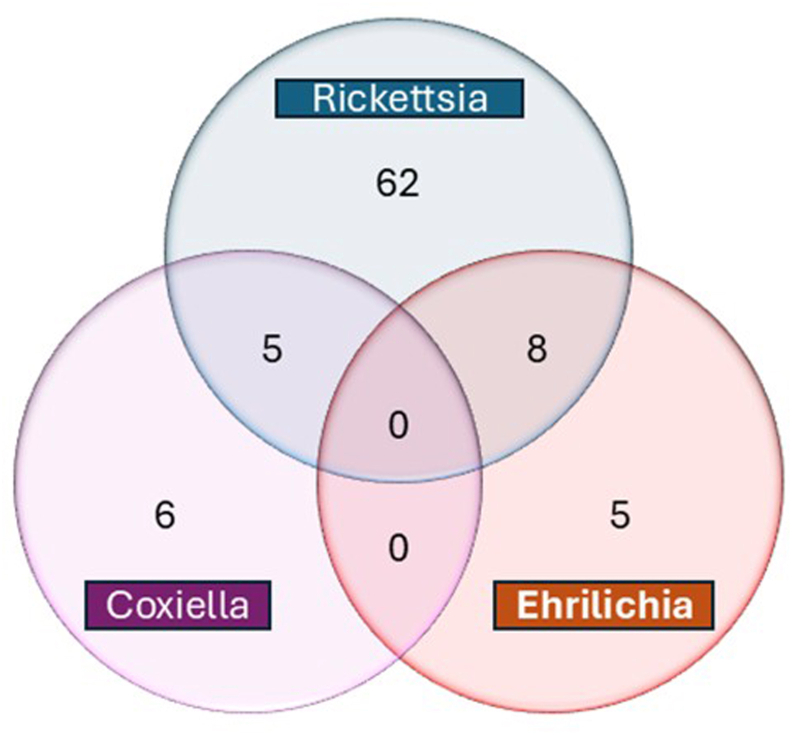
Venn diagram showing the distribution of single and co-infections with *Rickettsia* spp., *Coxiella burnetii*, and *Ehrlichia* spp. in tick samples, irrespective of tick species and collection site. Numbers represent the frequency of positive cases in each category.

Fourteen pools (10.3%) were co-infected with two pathogens. As shown in the Venn diagram (Fig. 5), eight pools (5.9%) were simultaneously infected with *Rickettsia* and *Ehrlichia* spp, all associated with *H. marginatum*. Five pools (3.7%) were co-infected with *Rickettsia* and *C. burnetii*, all from Jiroft County, including three *R. linnaei* pools and two *H. marginatum* pools. No pools were co-infected with *C. burnetii* and *Ehrlichia*, and no triple infections were detected.

Co-infection with *C. burnetii* and *Rickettsia* occurred exclusively in Jiroft (p = 0.002). While *H. marginatum* showed a higher likelihood of co-infection with *C. burnetii* and *Rickettsia* compared to *R. linnaei* (OR = 6.63), this association was marginally non-significant (p = 0.054) (Table 4, Table 5).

**Table 4:** Descriptive and Univariate Analysis of *Coxiella burnetii*, *Rickettsia* spp., and *Ehrlichia* spp. Infections Across Tick Genera and Collection Sites in Kerman Province, Iran, 2022.

		*Coxiella spp.*			*Rickettsia spp.*			*Ehrlichia spp.*		
		No (%)	OR (95%CI)	P value	No (%)	OR (95%CI)	P value	No (%)	OR (95%CI)	P value
**Tick**	***Hyalomma marginatum***	3(2.8)	0.07 (0.02-0.30)	<0.001	65 (60.2)	2.93 (0.86- 10.01)	0.03	12(11.1)	3.41 (0.42- 27.45)	0.47
	***Rhipicephalus linnaei***	8(29.6)			10(37.0)			1(3.7)		
County	Sirjan	0(0)	NA	0.001	31(66.0)	1	0.17	2(4.3)	1.000	0.24
	Baft	0(0.0)			26(53.1)	0.58 (0.26-1.33)		7(14.3)	3.75(0.73-1.08)	
	Jiroft	11(28.2)			18(46.2)	0.44 (0.19-1.06)		4(10.3)	2.57(0.45-14.86)

**Table 5:** Descriptive and Univariate Analysis of Co-infection with *Rickettsia* spp., *Coxiella burnetii*, and *Ehrlichia* spp. Across Tick Genera and Collection Sites in Kerman Province, Iran, 2022.

		*Coxiella* + *Rickettsia*			*Rickettsia* + *Ehrlichia*		
		No (%)	OR (95%CI)	P value	No (%)	OR (95%CI)	P value
**Tick**	***Hyalomma marginatum***	2(1.9)	6.63 (1.05-41.85)	0.054	8 (7.4	NA	0.36
	***Rhipicephalus linnaei***	3 (11.1)			0 (0.0)		
County	Sirjan	0 (0.0)	NA	0.002	2 (4.3)	1	0.27
	Baft	0 (0.0)	NA		1 (2.6)	2.56 (0.47- 13.88)	
	Jiroft	5 (12.8)	NA		8 (5.9)	0.59 (0.05-6.79)	

#### 3.2.1 Phylogenetic analysis

In the present study, PCR analysis confirmed the presence of *Rickettsia gltA* (834 bp) in 18 *Rickettsia* positive samples.

**Fig. 6:**
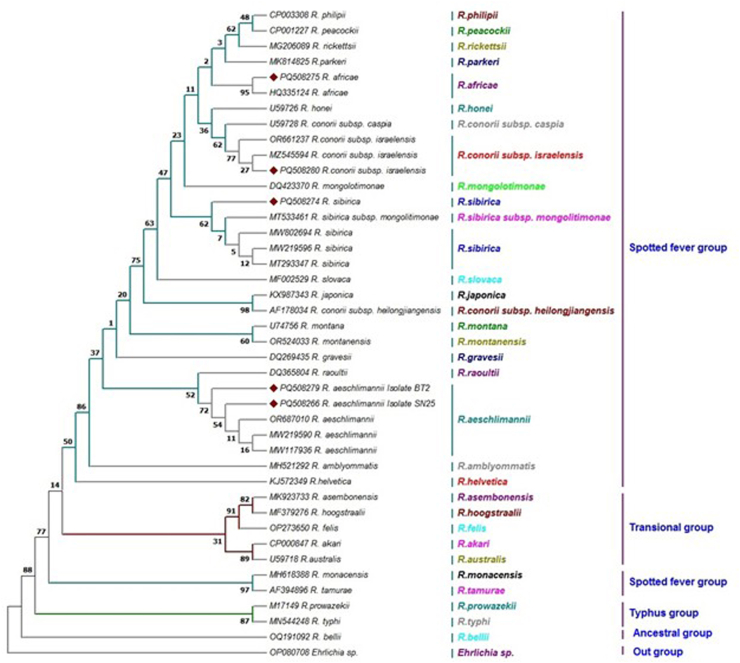
The maximum likelihood tree deduced from 834 bp of the *glt*A gene presents the phylogenetic position of *Rickettsia* spp. taxa under study among other sequences retrieved from GenBank. Colors reflect the different statuses of the species in each group. Only five representatives of the 18 sequences determined in this study (red diamonds) are included in the tree. One sequence of *Ehrlichia* spp. (OP060708) was used as an outgroup.

The identified species included 13 *R. aeschlimannii*, one *R. conorii subsp. israelensis*, one *R. africae,* and three *R. sibirica*. Phylogenetic analysis, based on the *gltA* gene and sequences retrieved from GenBank, delineated 24 distinct clades among the *Rickettsia* spp. Two representative *R. aeschlimannii* isolates were examined: BT2 (PQ508279) from Baft County and SN25 (PQ508266) from Sirjan County. These isolates exhibited 100% sequence similarity and query cover with *R. aeschlimannii* (OR687010), isolated from *H. marginatum* ticks in Russia. Both the isolates belonged to the *R. aeschlimannii* clade within the spotted fever group. Phylogenetic analysis demonstrated a close evolutionary relationship between this *Rickettsia* species and *R. raoultii* (Fig. 6) [39].

The three *R. sibirica* species identified in Sirjan County exhibited 100% sequence identity whit *R. sibirica* (MW219595), isolated from *H. dromedari* in Iran. Phylogenetic analysis revealed that these isolates belonged to the *R. sibirica* clade, closely related to *R. sibirica* subsp*. mongolitimonae* (MT533461), both of which belong to the spotted fever group (Fig. 6).

**Fig. 7:**
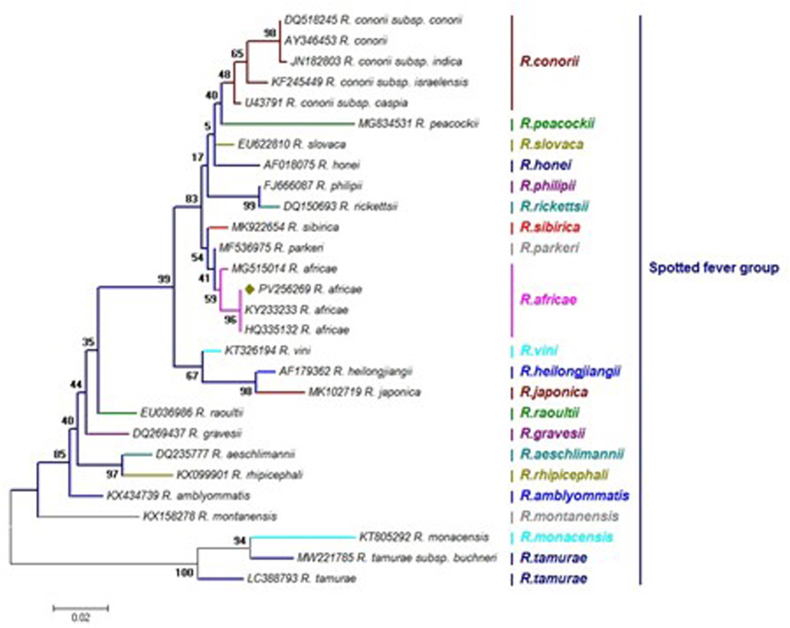
The maximum likelihood tree deduced from 632 bp of the *ompA* gene presented the phylogenetic position of *Rickettsia africa* taxa under study among other sequences retrieved from GenBank. Colors reflect the different statuses of the species in each genus. Only one representative of sequence determined in this study (olive diamonds) was included in the tree. This sample corresponds to the only positive case of *Rickettsia africana* isolated from *Hyalomma marginatum marginatum* in Sirjan city.

The sole *R. africae* strain identified in this study (PQ508275) exhibited 100% sequence identity with *R. africae* (HQ335124), previously isolated from *H. impeltatum* in Egypt. Both belong to the *R. africae* species and the spotted fever group. To further confirm the identity of *R. africana*, we sequenced the *ompA* gene in addition to the *gltA* gene for this sample. The *ompA* sequence was deposited in GenBank under accession number PV256269. BLAST analysis revealed 100% similarity to *R. africana* sequences previously isolated from *H. anatolicum* in Lebanon (KY233233) and *H. marginatum* in Egypt (HQ335132) (Fig. 6, Fig. 7).

The sole *R. conorii* subsp*. israelensis* identified in this study (PQ508280) exhibited 100% sequence identity with the *R. conorii* subsp. *israelensis* (MZ545594), isolated from human serum in Iran. Both belong to the *R. conorii* subsp*. israelensis* species and spotted fever group (Fig. 6).

**Fig. 8:**
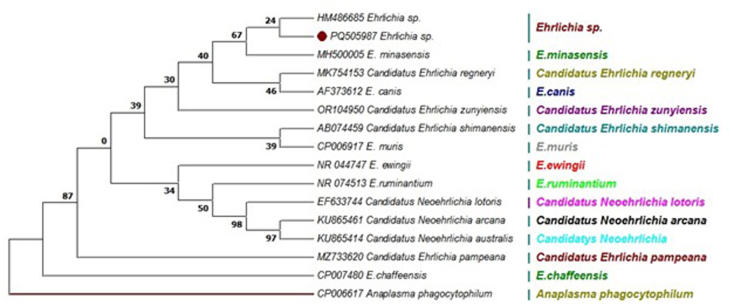
The maximum likelihood tree deduced from 555 bp of the *16S rRNA* gene presented the phylogenetic position of *Ehrlichia* spp. taxa under study among other sequences retrieved from GenBank. Colors reflect the different statuses of the species in each genus. Only one representative of sequence determined in this study (red circle) was included in the tree. one sequence of *Anaplasma phagocytophilum* (CP006617) was set as outgroup.

Additionally, The *Ehrlichia 16S rRNA* gene (555 bp) was detected in one of the *Ehrlichia* positive samples. The positive *Ehrlichia* spp. sample were isolated from *H. marginatum marginatum* and *Rh. linnaei*. Phylogenetic analysis based on the *16S rRNA* gene and sequences from GenBank, which were divided into 12 clades, revealed 100% sequence identity between this sample and *Ehrlichia* spp. (KJ410250) isolated from *H. asiaticum* in China. This sample clustered within the *Ehrlichia* spp. clade, above the *E. mianensis* clade (Fig. 8).

## 4 Discussion

This study presents the first comprehensive survey of tick-borne pathogens in domestic animals in Kerman province, Iran. *H. marginatum marginatum* was identified as the dominant tick species, followed by *Rh. linnaei*. Molecular analysis revealed a high prevalence of tick-borne pathogens, with 63.7%; (95% CI: 55.6%–71.8%) of the 135 tick pools testing positive for at least one pathogen. Among these, *Rickettsia* spp. and *Ehrlichia* spp. were the most prevalent, detected in 55.5% and 9.6% of tick pools, respectively. Other identified pathogens included *C. burnetii*, and *Bartonella* spp. notably, no infections with *F. tularensis* or *Borrelia* spp. were detected. Also, *R*.*aeschlimannii*, *R. sibirica*, *R. conorii* subsp. *israelensis* and *R. africae* were identified based on sequencing and phylogenetical analysis. Co-infections were observed in 10.3% of pools, including 5.9% (95% CI: 5.2%–15.5%) co-infected with *Rickettsia* spp. and *Ehrlichia* spp., 3.7% with *Rickettsia* spp. and *C. burnetii*, and 0.7% with *Rickettsia* spp. and *Bartonella* spp. These findings are consistent with previous studies conducted in Iran and other regions that have reported similar pathogenic profiles [9,12,[40], [41], [42]]. The increasing prevalence of tick-borne diseases can be attributed to the expansion of tick populations and their geographic distribution. Ticks serve as vectors for numerous pathogens, posing significant health risks to humans, domestic animals, and wildlife [43].

The frequent movement of livestock across Iran and neighboring regions facilitates the dispersal of ticks and their associated pathogens, including *Rickettsia* spp., *C. burnetii*, and *Ehrlichia* spp., as well as co-infections involving these pathogens. In this study, the identification of infected ticks actively feeding on livestock underscores their potential role in transmitting these pathogens to domestic animals. Further studies integrating molecular, serological, and ecological approaches are required to better understand the dynamics of tick-borne disease transmission in Iran.

The ecological adaptability of *H. marginatum*, enabling them to thrive in diverse environments, contributes to their significant role in the transmission of tick-borne diseases [[44], [45], [46], [47], [48]]. This species is a primary vector of Crimean-Congo hemorrhagic fever virus (CCHFV) [49,50] *R. aeschlimannii* [[51], [52], [53], [54]] and *Ehrlichia* spp. in Eurasia [55]. In Iran, *H. marginatum* is widely distributed and active during spring and autumn, infesting a range of hosts, including cattle, horses, and various wildlife species. Its ability to adapt to diverse climatic conditions and habitats, coupled with its role as a vector for multiple pathogens, poses a substantial threat to both animal and human health [56]. In this study, we also identified *H. marginatum* as a vector for *C. burnetii* and *Bartonella* spp.

The brown dog tick, *Rh. linnaei*, is the most widespread tick species globally. Ethologically, *Rh. linnaei* is an endophilic, monotropic, three-host tick, primarily infesting dogs but also capable of parasitizing other hosts, including humans. While it is highly endophilic, *Rh. linnaei* can also survive in outdoor environments, provided suitable refuges are available. This adaptability highlights its ability to employ diverse survival strategies [57]. Additionally, *Rh. linnaei* serves as a vector for several disease agents, including *C. burnetii*, *E*. *canis*, *R. conorii*, and *R. rickettsii*, many of which pose zoonotic risks. In this study, we confirm the role of *Rh. linnaei* as a vector for *Rickettsia* spp., *C. burnetii*, and *Ehrlichia* spp., consistent with previous findings [58].

This study demonstrated a high prevalence (55.5%) of *Rickettsia* spp. in the tested tick samples, with four distinct species identified. Among positive sample for *Rickettsia* spp., *R. aeschlimannii* was the most dominant, accounting for 72.2%. These findings are consistent with previous studies conducted in Iran, such as the 53.6% prevalence reported in ticks infesting small ruminants in western Iran and the 25.2% prevalence in ticks collected from stray dogs in the northern region [59]. However, the prevalence observed in this study is markedly higher than previous reports from Kerman Province, where earlier studies detected *Rickettsia* in only 24.3% of ticks from domestic animals and 4% of ticks from stray dogs [9,42]. The observed variation in prevalence may be attributed to differences in ecological conditions, host availability, and sampling methodologies. Climatic factors, particularly temperature and humidity, influence tick distribution and pathogen circulation, potentially contributing to regional differences in *Rickettsia* prevalence. Furthermore, the high infection rate in this study underscores the potential role of tick species in the epidemiology of rickettsial diseases, emphasizing the need for enhanced vector surveillance and molecular characterization of circulating strains. Given the zoonotic potential of *Rickettsia* spp., particularly *R. aeschlimannii*, our findings highlight the importance of active monitoring and control strategies to mitigate the risks associated with tick-borne rickettsioses in both animal and human populations.

The *Ehrlichia* spp. was not definitively identified in this study, and its prevalence in *Rh*. *sanguineus* was considerably lower than in *Hyalomma* spp. This lower prevalence contrasts with reports from other regions of the country [41,[60], [61], [62], [63]]. These differences in tick infestation rates could be related to several factors, including sampling methods, diagnostic methods, season, tick species, and, tick feeding status [41].

The higher prevalence of *C. burnetii* in *Rh. linnaei* compared to *H. marginatum* is consistent with previous findings from Kerman [9,64]. This finding reinforces the role of *Rh. linnaei* as an important vector in the transmission of *C. burnetii* and underscores the need for targeted surveillance and control measures to mitigate the risk of *Q fever* in both human and animal populations.

Collectively, these findings, coupled with previous research, indicate that various *Hyalomma* spp. and *Rhipicephalus* spp. may serve as vectors for pathogens transmitted to cattle, goats, dogs, and sheep in different regions of Iran. In addition to *Hyalomma* spp., other tick species, such as *R. turanicus*, *Rh*. *sanguineus* s.l., *Dermacentor marginatus*, and *Haemaphysalis concinna*, have been identified as confirmed or potential vectors for diverse bacterial pathogens within the country [9,10,41].

The low prevalence of *Ehrlichia* spp. in non-engorged ticks suggests that transovarial transmission of this bacterium may be minimal or absent in *Hyalomma* spp. and *Rhipicephalus* spp. [48]. In contrast, transovarian transmission has been documented in *Rickettsia* spp. Although transstadial transmission is well established for tick-borne rickettsiae, the role of transovarial transmission, especially in natural environments, remains understudied [48].

*R. aeschlimannii* was first isolated from a traveler returning from Morocco to France [65], and subsequently from *H. marginatum* in Morocco [39]. A wide range of hard ticks have been identified as potential vectors for this species in the Eastern Mediterranean region [12]. Our findings corroborate and expand on the established role of *H. marginatum* as a vector, underscoring its epidemiological significance in the transmission of *R. aeschlimannii*. Moreover, human infections associated with this pathogen have been reported in regions such as Tunisia and Morocco, thereby emphasizing its zoonotic potential [66,67]. The emergence of *R. aeschlimannii* as a potential human pathogen, coupled with increased global travel and trade, raises concerns about its potential geographic expansion.

*R. sibirica*, the causative agent of North Asian tick typhus, was first identified in *H. asiaticum* in Mongolia in 1991 [65]. Over the years, its presence has been confirmed in multiple *Hyalomma* species, further highlighting the adaptability and broad vector range of this pathogen [12]. Notably, the first human case of *R. sibirica* was documented in France in 1996, underscoring its zoonotic potential and risk of cross-border transmission [68]. Although studies in Cyprus and Iran have associated *H. excavatum* and *D. marginatus,* respectively, with *Rickettsia* infections [10,54], our research provides strong evidence that *H. marginatum* is a significant vector of *R. sibirica*. This finding aligns with other reports from the Middle East, where *R. sibirica*-positive *Hyalomma* spp. were collected directly from humans [69].

*R. conorii* is a well-known causative agent of Mediterranean spotted fever, which is primarily transmitted by *Rh. linnaei* [70]. However, emerging evidence suggests a broader vector spectrum for this pathogen [12]. In this study, we identified *H. marginatum* as a potential vector for *R. conorii subsp. israelensis* in Iran. This subspecies, endemic to the Mediterranean region, southern Africa, and parts of Europe, has been increasingly recognized as an emerging pathogen [71]. Although *R. turanicus* is a known vector for *R. conorii* in domestic animals in western Iran [10], the increasing number of human cases underscores the need for further investigation into alternative vectors [72,73], such as *H. marginatum*. Our findings highlight the importance of expanded surveillance to better understand the epidemiology of *R. conorii* infections in Iran.

*R. africana* was initially isolated from a febrile patient in Zimbabwe [74]. Although *Amblyomma variegatum* and *A. hebraeum* are recognized as primary vectors [75], a wider range of hard ticks can transmit this pathogen [12]. In this study, *H. marginatum* was identified as a potential vector of *R. africana* for the first time in Iran, marking the first report of this *Rickettsia* species outside the African continent. Although no human cases have been documented in the Eastern Mediterranean region, animal infections have been reported. The presence of potential reservoir hosts such as dogs, camels, and mice underscores the need for further research into alternative transmission routes and their role in human infections [12]. Accurate and timely diagnosis of *R. africana* infections in both humans and animals is crucial for effective disease management. Expanding surveillance efforts and developing advanced diagnostic tools are essential for addressing this emerging health threat.

The molecular analysis revealed four predominant tick-borne pathogens in the studied region: *Rickettsia* spp, *C. burnetii*, *Bartonella* spp, and *Ehrlichia* spp. The high prevalence of *Rickettsia* spp. among ticks in Sirjan County indicates a potential public health concern regarding tick-borne spotted fever transmission among local residents. Notably, multiple *Rickettsia* spp. was identified, suggesting substantial genetic diversity within the local tick population. Further investigation of ecological factors influencing pathogen distribution across Iranian regions is warranted to inform evidence-based public health interventions and optimize tick control strategies.

Given the zoonotic potential of the detected pathogens, implications for human health are important for high-risk people such as livestock owners, farmers, veterinarians, and people living in close contact with domestic animals. Accurate and timely diagnosis of tick-borne bacterial infections in both humans and animals is crucial for effective disease management. Expanding surveillance efforts and developing advanced diagnostic tools are essential for addressing this emerging health threat. Further investigation of ecological factors influencing pathogen distribution across Iranian regions is warranted to inform evidence-based public health interventions and optimize tick control strategies. It is imperative that at-risk individuals be educated about these tick-borne pathogens. The health system should also be trained on the presence of these pathogens and the possibility of these diseases so that potential patients can be identified and treatment in a timely manner.

## 5 Conclusion

This study highlights the prevalence of *Rickettsia* spp. and *Ehrlichia* spp. infections in Kerman Province. The identification of multiple *Rickettsia* spp., including *R. ashyamanii*, *R. conorii* subsp. *israelensis*, *R. siberica*, and *R. africana*, underscores the diverse range of tick-borne pathogens circulating in the region. These findings emphasize the importance of public health measures for preventing tick-borne diseases and protecting the local population.

## Funding

The study was funded by the Pasteur Institute of Iran (Research Centre for Emerging and Reemerging Infectious Diseases) and Shahid Bahonar University of Kerman.

## CRediT authorship contribution statement

**Shahin seidi:** Methodology, Software, Writing – original draft. **Mina Latifian:** Methodology, Writing – review & editing. **Safoura Moradkasani:** Methodology, Writing – original draft. **Ali Ghorbani:** Methodology, Writing – review & editing. **Mina Arabian:** Methodology. **Mohammad khalili:** Data curation, Writing – review & editing. **Saber Esmaeili:** Project administration, Writing – review & editing.

## Declaration of competing interest

The authors declare that they have no known competing financial interests or personal relationships that could have appeared to influence the work reported in this paper.

## Data Availability

The data that support the findings of this study are available from the corresponding author upon reasonable request.
